# Echocardiographic description and outcomes in a heterogeneous cohort of patients undergoing mitral valve surgery with and without mitral annular disjunction: a health service evaluation

**DOI:** 10.1186/s44156-022-00004-7

**Published:** 2022-07-13

**Authors:** Sadie Bennett, Jacopo Tafuro, Marcus Brumpton, Caragh Bardolia, Grant Heatlie, Simon Duckett, Paul Ridley, Prakash Nanjaiah, Chun Shing Kwok

**Affiliations:** 1 Heart and Lung Centre, University Hospitals of North Midlands, Stoke-on-Trent, ST4 6QG UK; 2 Keele University, Stoke-on-Trent, UK

**Keywords:** Mitral valve surgery, Mitral annular disjunction, Clinical outcomes

## Abstract

**Background:**

Mitral annular disjunction (MAD) is a structural abnormality characterized by the distinct separation of the mitral valve annulus/left atrium wall and myocardium. Little is known about the significance of MAD in patients requiring mitral valve surgery. This evaluation evaluates the echocardiographic characteristics and patient outcomes for patients with and without MAD who require mitral valve surgery.

**Methods:**

All patients who underwent mitral valve surgery and who had a pre-surgical transthoracic echocardiogram between 2013 and 2020 were included. Patient demographics and clinical outcomes were collected on review of patient electronic records.

**Results:**

A total of 185 patients were included in the analysis of which 32.4% had MAD (average MAD length 8.4 mm). MAD was seen most commonly in patients with mitral valve prolapse and myxomatous mitral valves disease (90% and 60% respectively). In the patients with MAD prior to mitral valve surgery, only 3.9% had MAD post mitral valve surgery. There were no significant difference in the severity of post-operative mitral regurgitation, arrhythmic events or major adverse cardiovascular events in patients with and without MAD.

**Conclusions:**

MAD is common in patients who undergo mitral valve surgery. Current surgical techniques are able to correct the MAD abnormality in the vast majority of patients. MAD is not associated with an increased risk of adverse clinical outcomes post mitral valve surgery.

**Supplementary Information:**

The online version contains supplementary material available at 10.1186/s44156-022-00004-7.

## Introduction

**Fig. 1:**
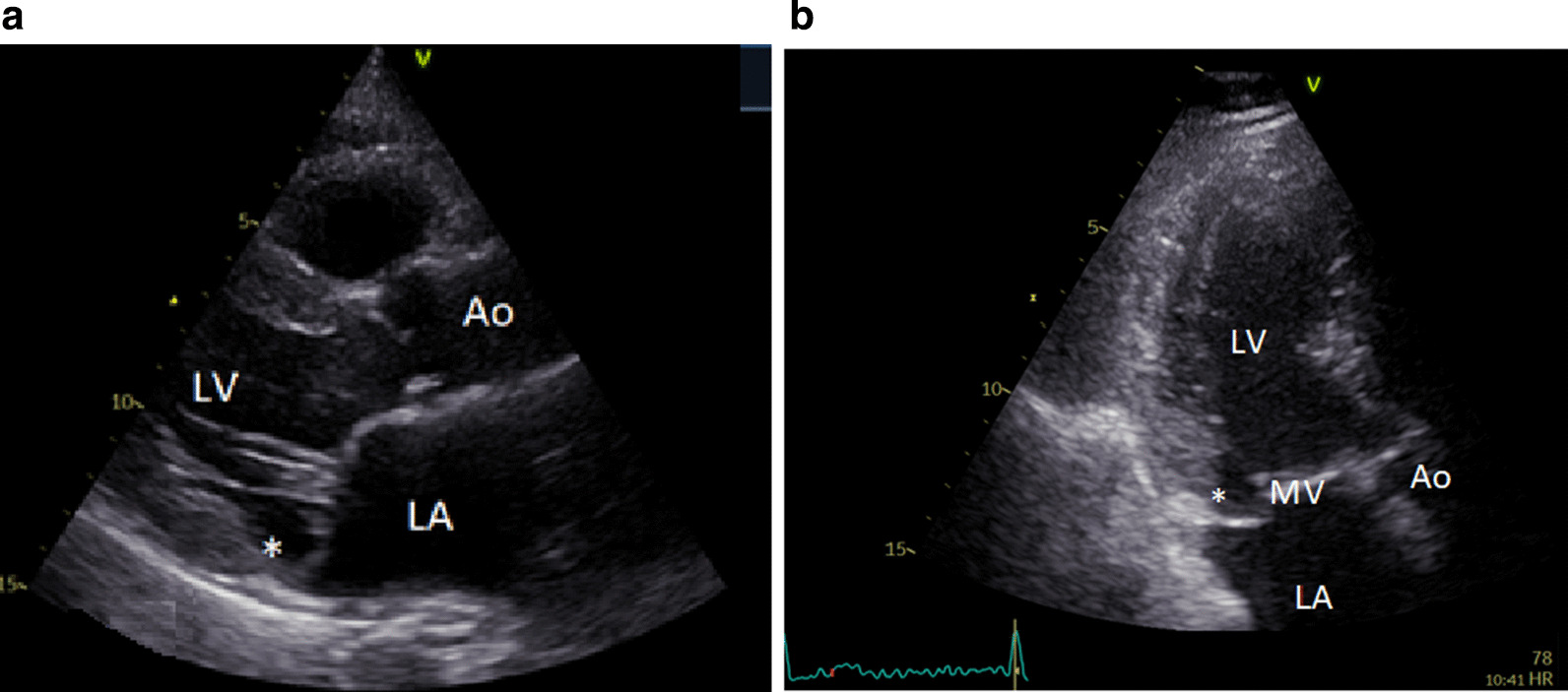
Transthoracic echocardiographic imaging of mitral annular disjunction. Mitral annular disjunction (*) as seen during ventricular on transthoracic echocardiography in the parasternal long axis view (**a**) and the apical three chamber view (**b**). *Ao* aorta, *LA* left atrium, *LV* left ventricle, *MV* mitral valve, *RV* right ventricle

**Fig. 2:**
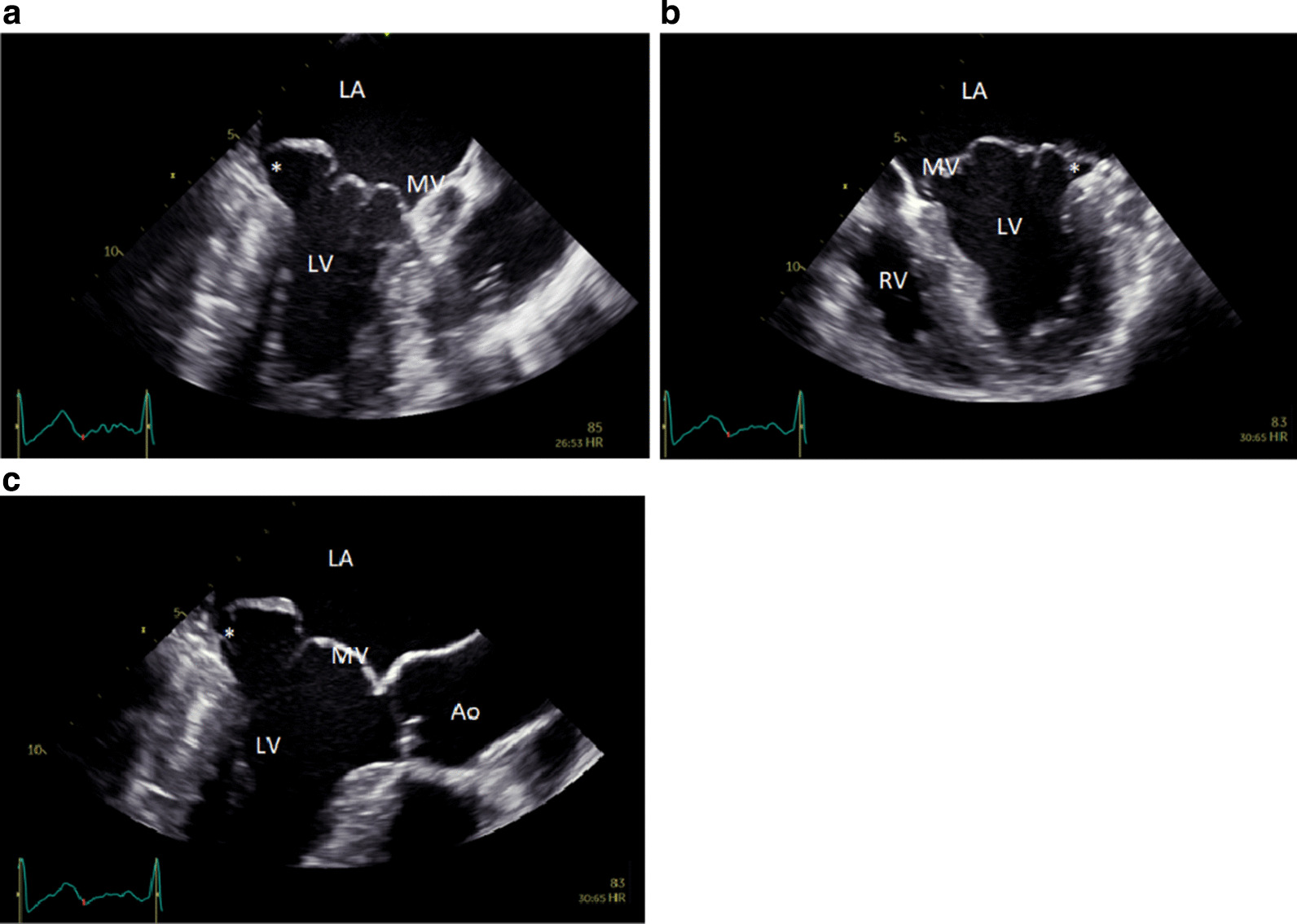
Transoesophageal echocardiography imaging of mitral annular disjunction. Mitral annular disjunction (*) as seen during ventricular on transoesophegeal echocardiography in the apical two chamber view (**a**), apical four chamber view (**b**) and apical three chamber view (**c**). *Ao* aorta, *LA* left atrium, *LV* left ventricle, *MV* mitral valve, *RV* right ventricle

Mitral annular disjunction (MAD) is a common structural abnormality defined by a distinct separation of the left atrium or mitral valve annulus and myocardium continuum (See Figs. 1 and 2) [1]. MAD is prevalent in patients with mitral valve disease particularly mitral valve prolapse [2]. It is thought to lead to paradoxical annular enlargement and annular flattening in ventricular systole which increases the stress placed on the mitral valve apparatus [3]. The hypermobility associated with MAD may also lead to increased left ventricular wall stress which may contribute to myocardial fibrosis, and left ventricular systolic impairment [4].

Mitral valve repair or mitral valve replacement in patients with severe mitral valve disease is associated with good long term patient outcomes [5, 6]. However, less favourable surgical outcomes have been reported in patients who have excessive mobility of the mitral leaflet or apparatus [7]. Echocardiography represent an effective method of evaluating MAD and mitral valve disease as this imaging modality has a good balance of temporal and spatial resolution. The lower cost and non-invasive approach of transthoracic echocardiography (TTE) compared to transoesophageal echocardiography makes it ideal to identify these structural abnormalities and monitor its progression. To date there is limited data how TTE can be used to detect MAD in patients who eventually undergo mitral valve surgery.

To understand the potential significance of MAD in this cohort of patients, we conducted a health service evaluation of patients who underwent TTE prior to mitral valve surgery. The aim of this evaluation was twofold; 1. To assess patient characteristics and the prevalence of MAD undergoing mitral valve surgery. 2. To assess the post surgical outcomes of patients with and without MAD including changes in MAD severity and adverse events of  in-hospital complications, arrhythmic events, cardiovascular events and mortality.

## Methods

This study was performed as part of an approved, registered (registration number: CA30321) and retrospective clinical audit as defined by our institution’s clinical audit and research department. Therefore ethical approval and patient consent was not required. The reporting of this study is in accordance with the strengthening the reporting of observational studies in epidemiology (STROBE) criteria [8].

### Study design

A retrospective observational evaluation was conducted of patients who underwent mitral valve surgery between 2013 and 2020 in the University Hospital of North Midlands cardiothoracic surgery database. Only patients who had a TTE pre surgery that could be reviewed were included.

### Data collection

The pre-surgical TTE images were reviewed by two experienced sonographers (JT and SB). The presence, location and extent of MAD, left ventricular ejection fraction and the severity of mitral regurgitation were all evaluated. For the patients with follow up TTE, post-surgical images were reviewed for left ventricular ejection fraction, right ventricular systolic function and presence, location and extent of MAD. Where multiple scans had been performed only the most recent scan was considered. MAD was defined as the separation of any distance between the attachment of the left atrial wall/mitral valve annulus and basal left ventricular myocardium at the end of ventricular systole. This was assessed in a 360-degree arc of the mitral valve annulus using the parasternal long axis, apical four, two and three chamber views on TTE. Left ventricular ejection fraction and right ventricular systolic function assessment was undertaken in accordance with national guidelines [9].

Mitral valve surgical procedural data, patient characteristics and patient comorbidities was retrieved from the mitral surgery procedural data was collected from cardiothoracic surgery database. Additional data on patient follow up, major adverse cardiovascular events (MACE) and cardiac arrhythmia outcomes were collected by MB and CB from our electronic patient records system. MACE included stroke, myocardial infarction or death which occurred during the follow-up period. Cardiac arrhythmia outcomes including documented evidence of atrial fibrillation, atrial flutter, ventricular tachycardia (sustained and non-sustained) and ventricular fibrillation.

### Statistical analysis

Statistical analysis was performed by CSK. The cohort was stratified into patients with and without MAD. Descriptive statistics were presented on patient demographics, comorbidities, aetiology of mitral valve disease, echocardiographic variables, surgical risk, operative variables. At follow-up the presence and severity of post-operative mitral regurgitation, arrhythmic events, major cardiovascular adverse events and mortality were also reported. The t-test and Fisher’s test was used for comparing difference between patients with MAD and no MAD for continuous and categorical variables, respectively.

## Results

There was a total of 558 patients who underwent mitral valve surgery between 2013 to 2020. In order to identify patients with MAD and those without MAD, we retrieved the TTE data however this was only available for 185 patients. Additional file 1: Table S1 shows the characteristics of the included patient cohort (n = 185) compared to the excluded patient cohort (n = 373). In the excluded patient cohort there were more males (p < 0.001), more mechanical valve replacements (p = 0.005). The duration of inpatient stay was slightly shorter for patients that were included in this evaluation compared to those that were excluded (p = 0.027).

As shown in Table 1, there were no statistical differences in terms of patient characteristics and comorbidities comparing patients with and without MAD. Mitral valve surgical intervention data can be seen in Table 2 with additional mitral valve operative data being shown in Additional file 1: Table S2. Mitral valve operative data specific to patients with MVP or myxomatous mitral valves can be seen in Additional file 1: Table S3. Duration of operation (285 ± 75 min vs 309 ± 122 min, p = 0.19) and post-operative hospital stay (11.9 ± 11.4 days vs 13.0 ± 10.9 days, p = 0.52) were similar amongst patients with and without MAD. The SCTS log Euroscore was significantly lower in patients with MAD than without MAD (4.5 ± 4.7 vs 7.5 ± 11.2, p = 0.043). Mitral valve surgical interventions included mitral valve repair (60.5%), mechanical (22.0%) and bioprosthetic (20%) mitral valve replacement.

**Table 1:** Patient characteristics and comorbidities

Variable	MAD (n = 60)	No MAD (n = 125)	p-value
Mean age (± SD)	66.7 ± 13.4	65.3 ± 13.8	0.51
Male	49 (81.7%)	87 (70.2%)	0.11
Smoking current or ex	25 (41.7%)	69 (55.2%)	0.12
Body mass index (± SD)	29.0 ± 23.9	26.1 ± 4.4	0.21
Hypertension	13 (21.7%)	35 (28.0%)	0.38
Hypercholesterolaemia	6 (10.0%)	20 (16.0%)	0.37
Diabetes mellitus	2 (3.3%)	9 (7.2%)	0.51
Angina	12 (20.0%)	34 (27.2%)	0.36
Ischaemic heart disease	7 (11.7%)	12 (9.6%)	0.80
Previous cardiac surgery	0 (0%)	1 (0.8%)	1.00
Previous PCI	3 (5.0%)	11 (8.9%)	0.55
Heart failure	2 (3.3%)	2 (1.6%)	0.60
Atrial fibrillation	13 (21.7%)	24 (19.2%)	0.70
Chronic lung disease	3 (5.0%)	10 (8.0%)	0.55
Stroke	0 (0%)	2 (1.6%)	1.00
Pulmonary hypertension	18 (30.0%)	52 (42.3%)	0.15
Renal disease	31 (51.7%)	72 (57.6%)	0.53
Creatinine (± SD)	81.6 ± 23.1	90.6 ± 44.7	0.14

**Table 2:** Mitral valve surgical intervention

Variable	Total (n = 185)	MAD (n = 60)	No MAD (n = 125)	p-value
*Operation type*				
Ring	112 (60.5%)	41 (68.3%)	71 (56.8%)	0.15
Biological	37 (20.0%)	11 (18.3%)	26 (20.8%)	0.85
Mechanical	41 (22.2%)	8 (13.3%)	33 (26.4%)	0.058
*Type of ring*				
Annuloplasty only	11 (9.8%)	3 (7.3%)	8 (11.3%)	0.74
Annuloplasty + leaflet repair	65 (58.0%)	29 (70.7%)	36 (50.7%)	0.048
Resection with neochords	23 (20.5%)	7 (17.1%)	16 (22.5%)	0.63
Replacement	14 (12.5%)	2 (4.9%)	12 (16.9%)	0.079
AF ablation	17 (9.2%)	7 (11.7%)	10 (8.0%)	0.68
Left atrial appendage closure/AtriClip	18 (9.7%)	7 (11.7%)	11 (8.8%)	0.81

Table 3 shows the TTE data for patients with and without MAD. The most common reason for mitral valve surgery was mitral valve prolapse and myxomatous mitral valve disease with moderate or severe mitral regurgitation. MAD was seen most commonly in patients with mitral valve prolapse (MVP) and myxomatous mitral valve (90% and 60% respectively). MAD was also associated with worse degrees of mitral regurgitation (moderate to severe) in comparison to patients without MAD (85.0% vs 68.0%, p = 0.044). In patients with MAD, the average length was 8.4 ± 3.9 mm. MAD was most commonly seen in the inferolateral left ventricular wall in comparison to other left ventricular regions (76.3% vs 23.7%) and was more commonly seen with posterior MVP in comparison to anterior MVP (85.0% vs 25.0%).

**Table 3:** Transthoracic echocardiographic data

Variable	MAD (n = 60)	No MAD (n = 125)	p-value
*MAD length (mm)*			–
Average ± SD	8.4 ± 3.9	–	
Median [IQR]	7 [5 to 11]	–	
*MAD location*		–	–
Infero-lateral	45 (76.3%)		
Other	14 (23.7%)		
*LVEF*			0.028
Average ± SD	60.1 ± 8.4%	57.1 ± 8.7%	
LV global impairment	5 (8.3%)	12 (9.6%)	1.00
Any RWMA	0 (0%)	12 (9.6%)	0.010
Impaired RV function	6 (10.0%)	18 (14.4%)	0.12
*MR severity*			0.044
Moderate or severe	51 (85.0%)	85 (68.0%)	
Ischaemic heart disease	0 (0%)	10 (8.0%)	0.032
Infective endocarditis	6 (10.0%)	19 (15.2%)	0.37
MVP	54 (90.0%)	91 (72.8%)	0.008
Anterior	15 (25.0%)	24 (19.2%)	0.005
Posterior	51 (85.0%)	72 (57.6%)	< 0.001
Barlows	3 (2.4%)	2 (3.3%)	0.66
Fail	14 (23.3%)	23 (18.4%)	0.44
Myxomatous MV	46 (60.0%)	49 (39.2%)	0.023
Restriction	0 (0%)	9 (7.2%)	< 0.001
Rheumatic	1 (1.7%)	1 (0.8%)	0.49
Post-op echo	48 (80.0%)	86 (68.8%)	0.20
Post-op residual MR	28 (46.7%)	52 (41.6%)	0.18
*MR severity post-op*			0.34
Mild or none	26 (43.3%)	39 (31.2%)	
Moderate	5 (8.3%)	15 (12.0%)	
Severe	1 (1.7%)	1 (0.8%)	
MAD post op echo	2 (3.9%)	(0%)	0.13
Follow up for post-op echo	834 ± 790	680 ± 693	0.24

p-value t-test or Fisher’s exact test

Of the 185 patients included in this evaluation, 89 patients had follow up ≥ 4 years with 42 patients having follow-up ≥ 6 years. TTE follow-up duration for patients with and without MAD was similar (834 ± 790 days vs 680 ± 693 days, p = 0.24). Among the patients with a post-operative TTE data, only 2 patients (3.9%) continued to show MAD post mitral valve surgery. Post-operative mitral regurgitation was seen in similar proportions of patients with and without MAD (46.7& vs 41.6%, p = 0.18). Reassuring, the vast majority of patients had mitral regurgitation that was graded as none or mild in severity (43.3% vs 31.2%, p = 0.32).

Long term follow-up for cardiac arrythmia and MACE rates can be seen in Table 4. Follow-up durations for MAD and non MAD patients was similar (1459 ± 881 days vs 1388 ± 783 days, p = 0.59). Over this follow-up period there was no significant difference in the event rates of atrial fibrillation (61.7% vs 59.2%, p = 0.59) or atrial flutter (6.7% vs 12.8%, p = 0.31) in patients with and without MAD respectively. Although not statistically significant, ventricular arrhythmias (including non-sustained ventricular tachycardia, sustained ventricular tachycardia and ventricular fibrillation) where only seen in patients without MAD. There was no significance difference in MACE in patients with and without MAD. During the follow-up duration a total of 40 patients died, causes of death can be seen in Additional file 1: Table S4. Survival analysis of patient with and without MAD can be seen in Additional file 1: Fig. S1.

**Table 4:** Long term arrhythmia and outcomes data

Post-operative events	MAD (n = 60)	No MAD (n = 125)	p-value
Follow up for mortality (years)	4.1 ± 2.3	3.9 ± 2.2	0.59
Atrial fibrillation	37 (61.7%)	74 (59.2%)	0.87
Atrial flutter	4 (6.7%)	16 (12.8%)	0.31
Non-sustained ventricular tachycardia	0 (0%)	3 (2.4%)	0.55
Ventricular tachycardia	0 (0%)	3 (2.4%)	0.55
Ventricular fibrillation	0 (0%)	5 (4.0%)	0.52
Stroke	3 (5.0%)	10 (8.0%)	0.55
Myocardial infarction	3 (5.0%)	6 (4.8%)	1.00
Death	28 (22.4%)	12 (20.0%)	0.85

## Discussion

Our retrospective study highlights that MAD is a common finding in patients requiring mitral valve surgery for any aetiology, with MAD occurring in approximately one third of patients. In this population, it is most common among patients with mitral valve prolapse and myxomatous mitral valve disease. Our results suggest that MAD does not affect surgical outcomes and surgery appears to correct the MAD in a vast majority of patients. In this population, we found no evidence of increased arrhythmic risk for patients with MAD compared to no MAD. These findings suggest that MAD is a frequent incidental finding in patients who undergo mitral valve surgery which does not impact patient outcomes.

We found that MAD is common in patients undergoing mitral valve surgery which has been described before. Eriksson et al. reported a much higher prevalence of MAD of 97% in advanced myxomatous mitral valve disease and 9% in mild to moderate myxomatous mitral valve disease [7]. The difference in rate in Eriksson et al. likely related to the difference in the evaluated population. The current study includes all aetiologies for mitral valve disease requiring surgery rather than advanced myxomatous mitral valve disease alone and the current study used TTE rather than transoesophageal echocardiography. Our study results are more consistent with the 38% rate of ≥ 5 mm MAD evaluated by TTE in a cohort of 64 patients with Barlow’s disease undergoing mitral valve surgery reported by Hiemstra et al [5] and the rate of 35% in 89 patients with mitral valve prolapse detected on cardiac magnetic resonance imaging described by Essayagh et al. [10]

To the best of our knowledge only one study by Eriksson et al. [7] has investigated patients with and without MAD pre and post mitral valve surgery. Whilst there were similarities between Eriksson et al. [7] and our evaluation in terms patient gender (66% male vs 72.2% male) and MAD length (10 ± 3 mm vs 8.4 ± 3.9 mm). It is difficult to make further comparisons as Erikson et al [7] only included patients with MVP or Barlow’s disease (32 with mild to moderate disease and 32 with advanced disease) who underwent mitral valve repair. The patient cohort studied in Eriksson et al. [7] were generally young (52 ± 12 years vs 65 ± 13.7 years) and had a lower incidence of requiring coronary artery bypass grafting at the time of mitral valve surgery (4.6% vs 19.8%).

This evaluation provides insight into the echocardiographic characterization of MAD. In term of location, it is most commonly seen in the inferolateral left ventricular myocardial wall but it can be observed in nearly 1 in 4 patients in other left ventricular regions. In addition, MAD was more frequently seen in patients with myxomatous mitral valve disease and MVP particularly involving the P2 scallop as also reported by Lee et al. [6] There is also a greater proportion of patients with moderate or severe mitral regurgitation with MAD compared to no MAD. Compared to patients without MAD, patients with MAD have significantly higher left ventricular ejection fraction as previously seen in Essayagh et al. [10] and Konda et al. [11]

The mitral valve pathology and impact of surgery on MAD merits consideration. Among patients with degenerative mitral regurgitation with a flail leaflet, MV repair is associated with lower operative mortality, better long-term survival and is an independent predictor of higher postoperative ejection fraction. In addition, the conservation of the mitral valve architecture is associated with a more favourable geometry and remodelling of the left ventricle after surgical correction of the regurgitation. The current finding that most patients post mitral valve surgery who had MAD pre-surgery no longer had MAD on follow up echocardiogram may suggest that MAD is corrected by the techniques presented in the current study including annuloplasty only, annuloplasty and leaflet repair, resection with neochords and valve replacement. More studies are needed to determine if indeed MAD may be corrected by stabilising the annulus only.

This study adds to the growing awareness of MAD as an incidental finding in patients and should be looked for in patients with MVP and myxomatous mitral valves. On TTE imaging, it can be seen in the parasternal long axis, apical four, two and three chamber views. The key to its identification is looking for it as it is only seen in ventricular systole. It is notable that it can be seen on other imaging modalities including transoesphageal echocardiography [7], cardiac magnetic resonance imaging [12] and computer tomography imaging [13]. As demonstrated in Mantegazza et al., it is likely that the improved spatial resolution of cardiac magnetic resonance and computed tomography imaging lead to higher MAD prevalence rates in comparison to TTE and transoesphageal echocardiography. [12]

The concern regarding ventricular arrhythmias with MAD has drawn recent interest. Van Wijngaarden et al. reported a higher frequency of ventricular arrhythmia in patients with MAD than without MAD in patients with MVP and moderate to severe mitral regurgitation (39% vs 20%, p ≤ 0.001) [14]. Similarly, the recent study by Essayagh et al. of 595 patients with isolated mitral valve prolapse found that after propensity score matching the patients with MAD are at increased risk of clinical arrhythmic events (HR 2.60 95%CI 1.87–3.62) but MAD was not linked to increased death within the first 10 years post-diagnosis [15]. Contrary to the findings of the current study was the finding that the link between MAD and arrhythmic events persisted with time-dependent surgery and was weaker after mitral surgery. The arrhythmia mechanism underlying MAD is largely unknown. It has been suggested that arrhythmias may be related to hypermobility of the associated MAD basal left ventricular region contributing to excessive mechanical stress on the mitral valve annulus resulting in myocyte hypertrophy, fibrosis and subsequent electrical instability resulting in ventricular arrhythmias [16]. In this study we found that patients with MAD had no increased risk of arrhythmias when compared to patient without MAD in a cohort of patients undergoing mitral valve surgery. This finding is consistent with the low rates of arrhythmic events and sudden death (3.8%) over a median of 20.3 years follow up that was reported by Konda et al [11] and the lack of increased risk of arrhythmias in patients with MAD who undergo transcatheter aortic valve replacement as described by Tsianaka et al. [17] We build on the literature with the novel finding that current mitral valve surgical techniques are able to correct the MAD and there is no increase in developing residual mitral regurgitation which requires re-intervention among patients with MAD.

A key consideration is what to do with patients with MAD when it is found. Our evaluation found that no changes need to take place in terms of surgical approach for patients with and without MAD. In terms of post-surgical outcomes, there has been a growth of recent literature regarding the association between MAD and ventricular arrhythmias. There is evidence to support the use of implantable cardioverter defibrillators (ICD) following these arrhythmic events as secondary prevention. In the current study many patients did not have ventricular arrhythmias even at follow up. This suggests there is insufficient evidence for primary preventative use of ICD devices. It is likely that only a proportion of patients with MAD have increased arrhythmic risk and it has been suggested that those with MAD length > 8.5 mm are at high risk [18]. However, in our cohort many patients (average MAD length: 8.4 ± 3.9 mm) there were very few arrhythmic events in the group with MAD. More studies are needed to determine if there is a certain subgroup of patients who are more likely to have ventricular arrhythmias and what should be done to manage these patients.

Our evaluation has several limitations. First, data collection was retrospective and observational which may have inherent biases. However, the data is from a real world setting making it generalizable to a population of patients requiring mitral valve surgery regardless of aetiology. Secondly, we acknowledge that a proportion of patients may have initially been from a different catchment area, or may have relocated to a different catchment area, which may have affected follow up data collection. Thirdly, our study found an unexpected result that the proportion of patients with Barlow’s valve was low. This may be related to a diagnostic issue rather than a true finding as the data was retrieved from our local cardiothoracic database and we were unable to ascertain how reliable the data is. Fourthly, our database of mitral valve surgery includes some patients that would have had the mitral valve disease as the primary problem while others may have coronary artery bypass grafting as their primary problem and mitral valve disease as a secondary problem. An important limitation is that the primary reason for surgery is not clear in our evaluation but it only affects a small portion of the cohort as only 19.5% had coronary artery bypass grafting. Finally, the mortality and ventricular arrhythmia data in the cohort may be more opportunistic based on our hospital records rather than systematic where the patients were contacted for follow up.

## Conclusion

MAD is common in patients who undergo mitral valve surgery. Current surgical techniques are able to correct the MAD abnormality in the vast majority of patients. MAD does not appear to be associated with an increased risk of adverse clinical outcomes post mitral valve surgery.

## Supplementary Information

**Additional file 1: Table S1.** Comparison of the characteristics of the included vs excluded patients. **Table S2.** Additional mitral valve operation data. **Table S3.** Mitral valve operative data for patients with mitral valve prolapse or myxomatous mitral valves only. **Table S4.** Causes of death at follow up. **Figure S1.** Survival analysis of patients with and without MAD.

## Data Availability

Not applicable.

## Abbreviations

ICD: Implantable cardioverter defibrillators
MACE: Major adverse cardiovascular events
MAD: Mitral annular disjunction
MVP: Mitral valve prolapse
TTE: Transthoracic echocardiography
