# Supplementary material for: Echocardiographic description and outcomes in a heterogeneous cohort of patients undergoing mitral valve surgery with and without mitral annular disjunction: a health service evaluation
Source: Echo Res Pract. 2022 Jul 13;9:4. doi: 10.1186/s44156-022-00004-7 (PMC9277900; doi:10.1186/s44156-022-00004-7)

**Table S1:** Comparison of the characteristics of the included vs excluded patients

| **Variable** | **Included patients**  **(n=185)** | **Excluded patients**  **(n=373)** | **p-value** |
| --- | --- | --- | --- |
| Mean age (±SD) | 65.6±13.7 | 66.5±11.9 | 0.46 |
| Male | 138 (71.2%) | 212 (57.1%) | <0.001 |
| Smoking current or ex | 95 (50.8%) | 171 (46.1%) | 0.32 |
| Body mass index (±SD) | 27.0±14.2 | 27.5±5.5 | 0.62 |
| Hypertension | 49 (26.2%) | 116 (31.3%) | 0.24 |
| Hypercholesterolaemia | 27 (14.4%) | 59 (15.9%) | 0.71 |
| Diabetes mellitus | 11 (5.9%) | 24 (6.5%) | 0.86 |
| Angina | 47 (25.1%) | 103 (27.8%) | 0.55 |
| Ischaemic heart disease | 19 (10.2%) | 57 (15.4%) | 0.12 |
| Previous cardiac surgery | 1 (0.5%) | 21 (5.7%) | 0.21 |
| Previous PCI | 14 (7.6%) | 27 (5.7%) | 0.76 |
| Heart failure | 4 (2.1%) | 16 (4.3%) | 0.23 |
| Atrial fibrillation | 36 (19.3%) | 104 (28.0%) | 0.030 |
| Chronic lung disease | 13 (7.0%) | 39 (10.5%) | 0.22 |
| Stroke | 3 (1.6%) | 9 (2.4%) | 0.76 |
| Pulmonary hypertension | 71 (38.4%) | 122 (34.4%) | 0.40 |
| Renal disease | 103 (55.1%) | 189 (50.9%) | 0.37 |
| Creatinine (±SD) | 81.6±23.1 | 90.6±44.7 | 0.15 |
| Log Euroscore (±SD) | 10.2±11.8 | 9.8±10.6 | 0.73 |
| SCTS Log Euroscore (±SD) | 6.5±9.6 | 6.2±8.7 | 0.70 |
| Euroscore 2 | 4.4±7.9 | 5.1±8.4 | 0.36 |
| Year of operation  2013  2014  2015  2016  2017  2018  2019  2020 | 7 (3.7%)  37 (19.8%)  19 (10.2%)  25 (13.4%)  23 (12.3%)  31 (16.6%)  24 (12.8%)  21 (11.2%) | 59 (15.9%)  62 (16.7%)  50 (16.2%)  44 (11.9%)  44 (11.9%)  46 (12.4%)  33 (8.9%)  23 (6.2%) | <0.001 |
| CABG at operation | 37 (19.8%) | 94 (25.3%) | 0.17 |
| Aortic surgery at operation | 5 (2.7%) | 4 (1.1%) | 0.17 |
| Operation  Ring  Biological  Mechanical | 96 (54.6%)  37 (21.0%)  43 (24.4%) | 150 (41.4%)  83 (22.9%)  129 (35.6%) | 0.010 |
| Duration of operation | 302±110 | 331±150 | 0.027 |
| Retheatre | 12 (6.5%) | 32 (8.6%) | 0.95 |
| New neurological problem | 12 (6.6%) | 17 (4.6%) | 0.53 |
| GI complication | 9 (5.1%) | 18 (5.0%) | 1.00 |
| Post operation length of stay | 12.7d±11.0 | 13.5±12.9 | 0.44 |
| Alive at follow up | 10 (5.4%) | 17 (4.6%) | 0.68 |

p-value t-test or Fisher’s exact test

**Table S2:** Additional mitral valve operation data.

| **Variable** | **Total**  **(n=185)** | **MAD**  **(n=60)** | **No MAD**  **(n=125)** | **p-value** |
| --- | --- | --- | --- | --- |
| Log Euroscore (±SD) | 10.3±11.8 | 7.5±6.3 | 11.6±13.5 | 0.028 |
| SCTS Log Euroscore (±SD) | 6.5±9.7 | 4.5±4.7 | 7.5±11.2 | 0.043 |
| Euroscore 2 | 4.5±8.0 | 2.8±2.5 | 5.3±9.5 | 0.050 |
| Year of operation  2013  2014  2015  2016  2017  2018  2019  2020 | 7 (3.8%)  36 (19.5%)  19 (10.3%)  25 (13.5%)  23 (12.4%)  31 (16.8%)  23 (12.4%)  21 (11.4%) | 1 (1.7%)  16 (26.7%)  11 (18.3%)  2 (3.3%)  3 (5.0%)  13 (21.7%)  7 (11.7%)  7 (11.7%) | 6 (4.8%)  20 (16.0%)  8 (6.4%)  23 (18.4%)  20 (16.0%)  18 (14.4%)  16 (12.8%)  14 (11.2%) | 0.002 |
| CABG at operation | 36 (19.5%) | 9 (15.0%) | 27 (21.6%) | 0.33 |
| Aortic surgery at operation | 5 (2.7%) | 2 (3.3%) | 3 (2.4%) | 0.66 |
| AF ablation | 17 (9.2%) | 7 (11.7%) | 10 (8.0%) | 0.68 |
| Left atrial appendage closure/AtriClip | 18 (9.7%) | 7 (11.7%) | 11 (8.8%) | 0.81 |

Among 17 patients, with AF ablation 15 had a recurrence of AF post procedure (88.2%).

**Table S3.** Mitral valve operative data for patients with mitral valve prolapse or myxomatous mitral valves only.

| **Variable in group with MVP** | **Total**  **(n=136)** | **MAD**  **(n=51)** | **No MAD**  **(n=85)** | **p-value** |
| --- | --- | --- | --- | --- |
| Operation  Ring  Biological  Mechanical | 84 (60.9%)  27 (19.6%)  27 (19.6%) | 36 (69.2%)  8 (15.4%)  8 (15.4%) | 48 (55.8%)  19 (22.1%)  18 (22.1%) | 0.30 |
| Type of Ring  Annuloplasty only  Annuloplasty+leaflet repair  Resection with neochords | 2 (2.4%)  60 (72.3%)  21 (25.3%) | 1 (2.8%)  28 (77.8%)  7 (19.4%) | 1 (2.1%)  32 (68.1%)  14 (29.8%) | 0.54 |
| **Variable in group with myxomatous mitral valve** | **Total**  **(n=79)** | **MAD**  **(n=33)** | **No MAD**  **(n=46)** | **p-value** |
| Operation  Ring  Biological  Mechanical | 48 (60.8%)  13 (16.5%)  18 (22.8%) | 23 (69.7%)  5 (15.2%)  5 (15.2%) | 25 (54.4%)  8 (17.4%)  13 (28.3%) | 0.35 |
| Type of Ring  Annuloplasty only  Annuloplasty+leaflet repair  Resection with neochords | 0 (0%)  33 (68.8%)  15 (31.3%) | 0 (0%)  18 (78.3%)  5 (21.7%) | 0 (0%)  15 (60.0%)  10 (40.0%) | 0.22 |

**Table S4:** Causes of death at follow up.

| **Cause of death** | **n** |
| --- | --- |
| Pneumonia | 2 |
| Heart failure | 2 |
| Bleeding | 2 |
| Cancer | 2 |
| Operative complication | 2 |
| Sepsis | 2 |
| Stroke | 1 |
| Subarachnoid haemorrhage | 1 |
| Aortic dissection | 1 |
| Infective endocarditis | 1 |
| Multiorgan failure | 2 |
| Unknown | 22 |
| Total | 40 |

The mean age of patients that died was 71.6±12.6 at time of the operation.

**Figure S1:** Survival analysis of patients with and without MAD


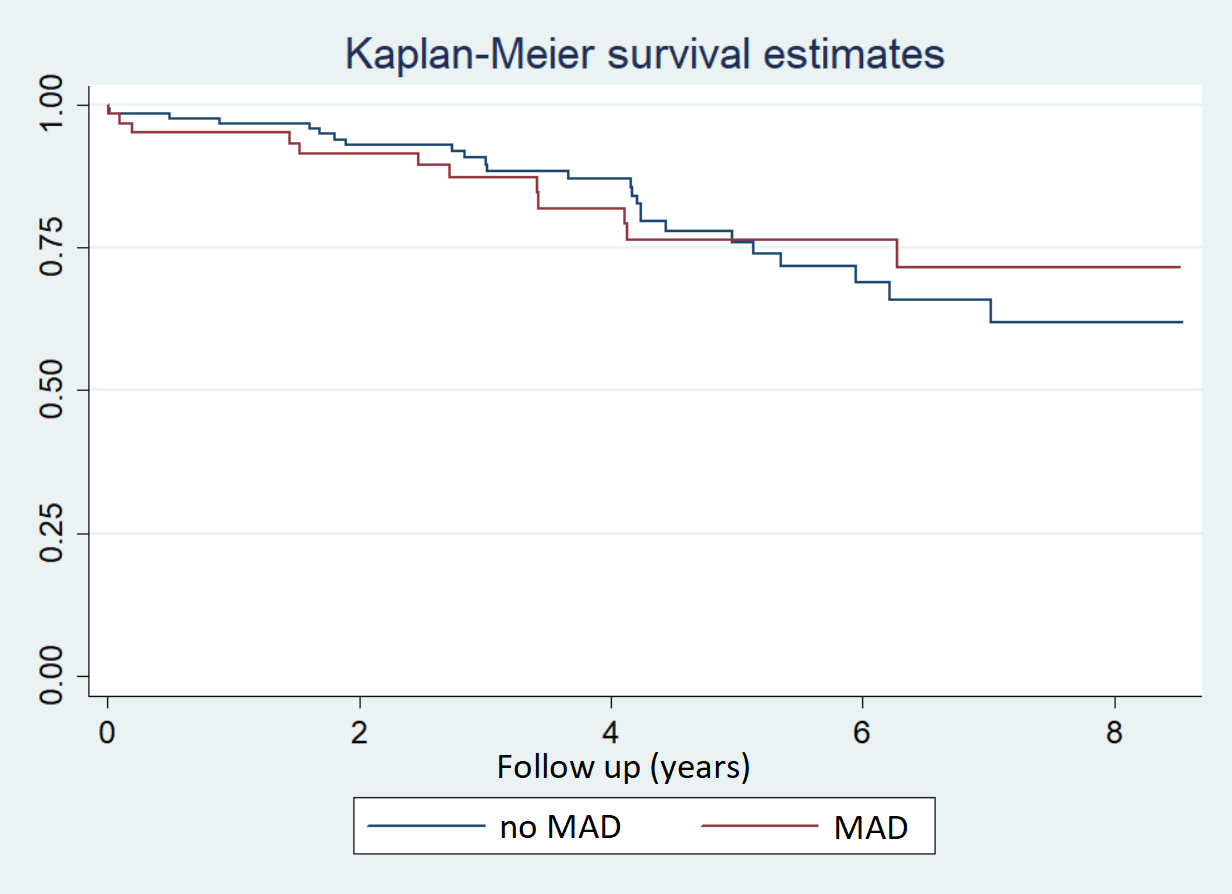

Supplement: Supplementary file 1 — Additional file 1: Table S1. Comparison of the characteristics of the included vs excluded patients. Table S2. Additional mitral valve operation data. Table S3. Mitral valve operative data for patients with mitral valve prolapse or myxomatous mitral valves only. Table S4. Causes of death at follow up. Figure S1. Survival analysis of patients with and without MAD. [file 44156_2022_4_MOESM1_ESM.docx]
